# Supplementary material for: Helicobacter pylori luxS mutants cause hyperinflammatory responses during chronic infection
Source: Microbiol Spectr. 2024 Dec 6;13(1):e01073-24. doi: 10.1128/spectrum.01073-24 (PMC11705807; doi:10.1128/spectrum.01073-24)
Supplement: Figure S1 — Gating strategy. [file spectrum.01073-24-s0001.pdf]

Supplemental Figure 1

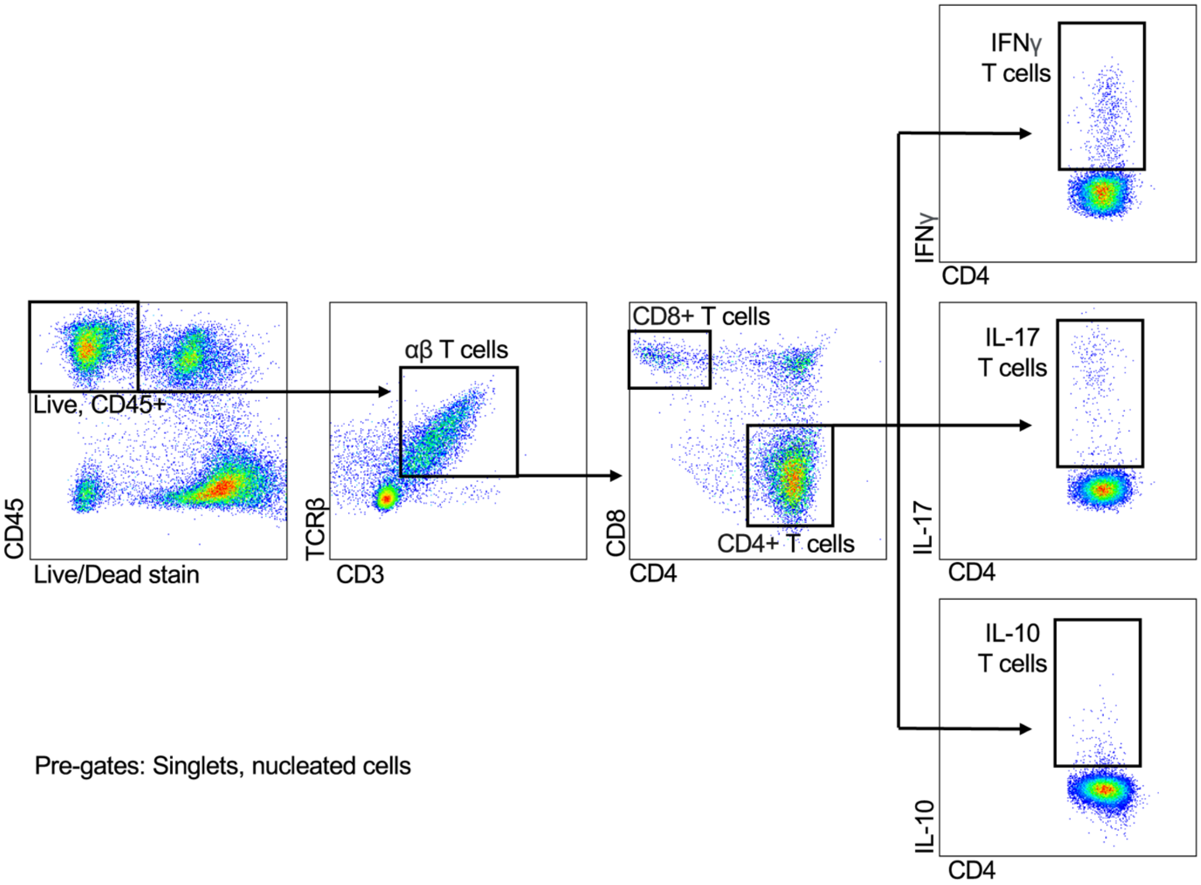

Supplemental Figure 1: Gating strategy used to identify effector and regulatory T cell populations by cytokine markers

Leukocytes were isolated from the corpus lamina propria via enzymatic digestion with collagenase IV and Percoll gradient centrifugation. Isolated leukocytes were analyzed via flow cytometry. Gating strategy for $CD4^{+}$ T cell populations by IFNγ (Th1), IL17a (Th17), or IL10 (Treg).
